# Supplementary material for: Transcriptomic analysis reveals differential regulation of synaptic components in pediatric and adult brain tumors
Source: bioRxiv. 2026 Oct 1:2026.09.30.755821. Preprint. [Version 1] doi: 10.64898/2026.09.30.755821 (PMC13648705; doi:10.64898/2026.09.30.755821)

bioRxiv preprint doi: https://doi.org/10.64898/2026.09.30.755821; this version posted October 2, 2026. The copyright holder for this preprint (which was not certified by peer review) is the author/funder, who has granted bioRxiv a license to display the preprint in perpetuity. It is made available under a CC-BY 4.0 International license.

Table Legends

Supplemental Table 1. Distribution of Tumor Samples Across Histopathological Categories
Summary of the number of tumor samples classified by histopathological subtype.

Supplemental Table 2. Catalogue of Synaptic Genes and Receptor Targets Analyzed in This Study
Comprehensive list of synaptic genes and receptor families included in the transcriptomic analyses.

Supplemental Table 3. Differential Gene Expression Profiles in Adult Glioma Subtypes Versus Non-Cerebellar Healthy Brain Tissue (GTEx)
Comparison of gene expression patterns between glioblastoma (GBM), astrocytoma, and oligodendroglioma samples and matched healthy brain data from the GTEx consortium.

Supplemental Table 4. Differential Gene Expression Profiles in Pediatric Brain Tumors Versus Non-Cerebellar Healthy Brain Tissue (GTEx)
Comparative transcriptomic analysis of pediatric high-grade glioma, diffuse intrinsic pontine glioma (DIPG), low-grade glioma, and ganglioglioma relative to non-cerebellar GTEx brain samples.

bioRxiv preprint doi: https://doi.org/10.64898/2026.09.30.755821; this version posted October 2, 2026. The copyright holder for this preprint (which was not certified by peer review) is the author/funder, who has granted bioRxiv a license to display the preprint in perpetuity. It is made available under a CC-BY 4.0 International license.

Supplemental Table 5. Differential Gene Expression Profiles in Medulloblastoma Molecular Subgroups Versus Cerebellar Tissue (GTEx)
Analysis of gene expression alterations in medulloblastoma subgroups (Group 3, Group 4, WNT, and SHH) relative to cerebellar samples from the GTEx database.

Supplemental Table 6. Differential Gene Expression Profiles in Ependymoma Subtypes Versus Non-Cerebellar Healthy Brain Tissue (GTEx)
Transcriptomic comparison of ependymoma subtypes against non-cerebellar healthy brain samples from GTEx.

Supplemental Table 7. Differential Gene Expression Profiles in Meningioma Versus Non-Cerebellar Healthy Brain Tissue (GTEx)
Comparison of gene expression in meningioma samples with corresponding non-cerebellar healthy brain tissue from GTEx.

bioRxiv preprint doi: https://doi.org/10.64898/2026.09.30.755821; this version posted October 2, 2026. The copyright holder for this preprint (which was not certified by peer review) is the author/funder, who has granted bioRxiv a license to display the preprint in perpetuity. It is made available under a CC-BY 4.0 International license.

Supplementary Figure 1

A

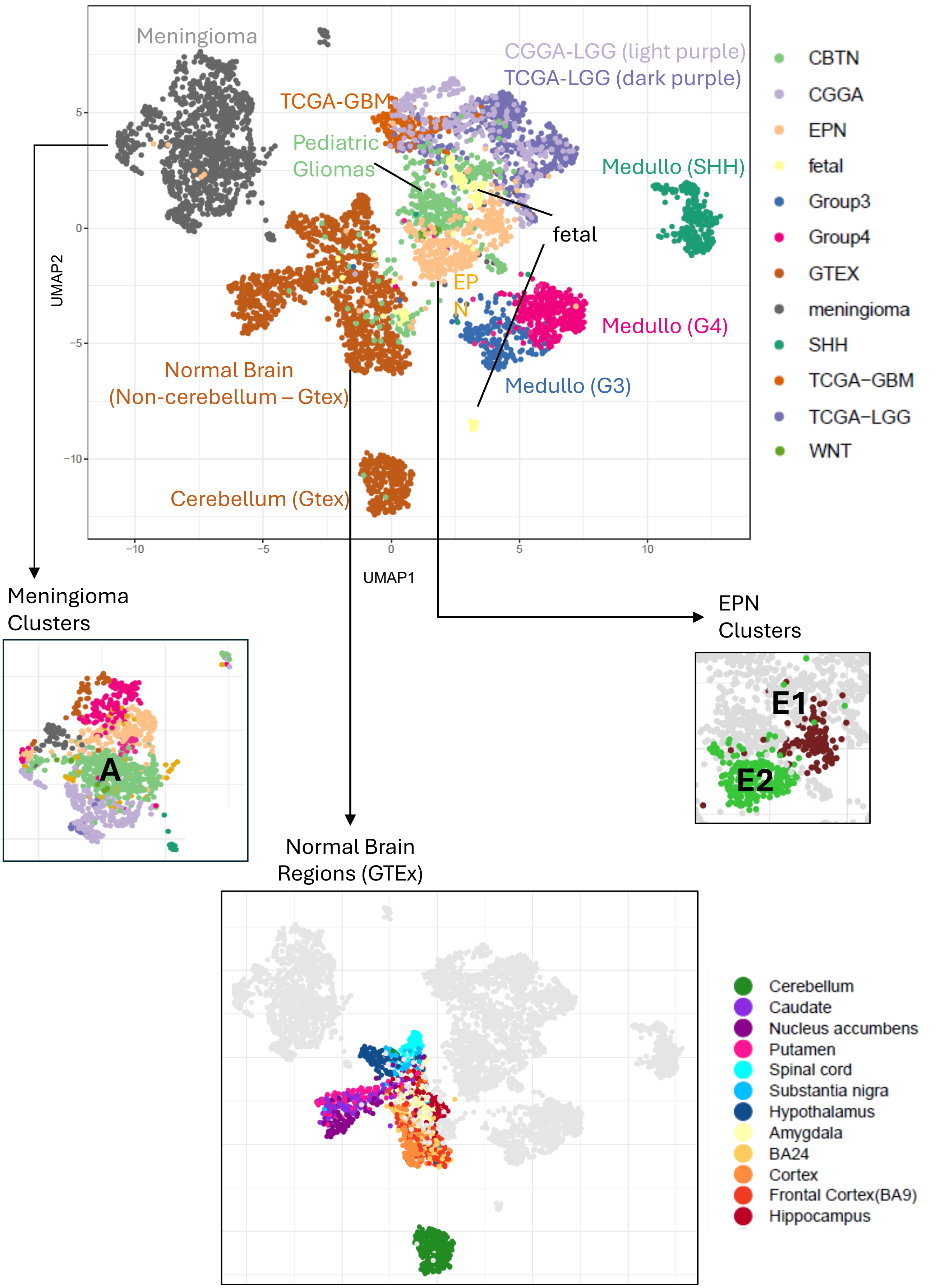

bioRxiv preprint doi: https://doi.org/10.64898/2026.09.30.755821; this version posted October 2, 2026. The copyright holder for this preprint (which was not certified by peer review) is the author/funder, who has granted bioRxiv a license to display the preprint in perpetuity. It is made available under a CC-BY 4.0 International license.

Supplementary Figure 2

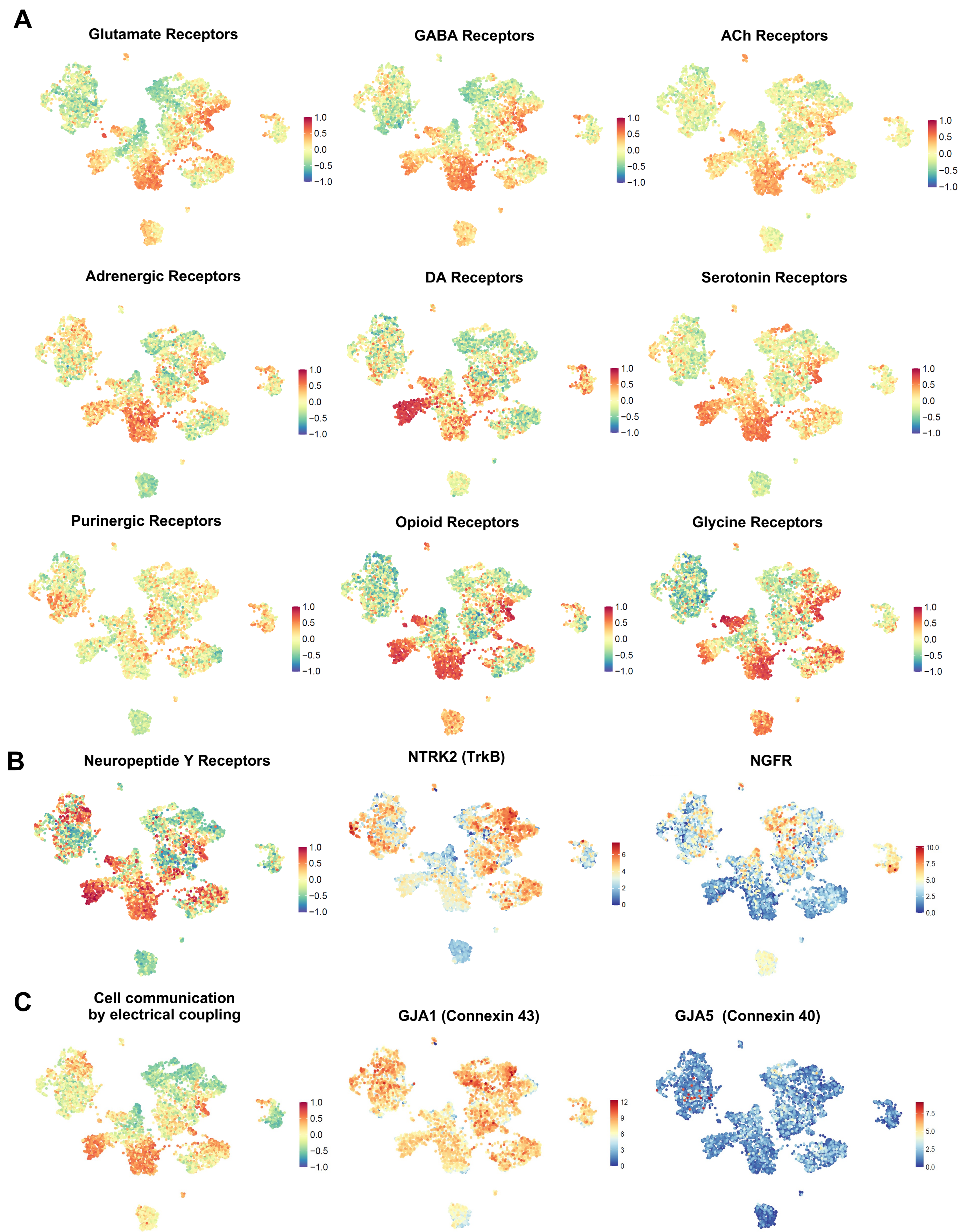

bioRxiv preprint doi: https://doi.org/10.64898/2026.09.30.755821; this version posted October 2, 2026. The copyright holder for this preprint (which was not certified by peer review) is the author/funder, who has granted bioRxiv a license to display the preprint in perpetuity. It is made available under a CC-BY 4.0 International license.

Supplementary Figure 3

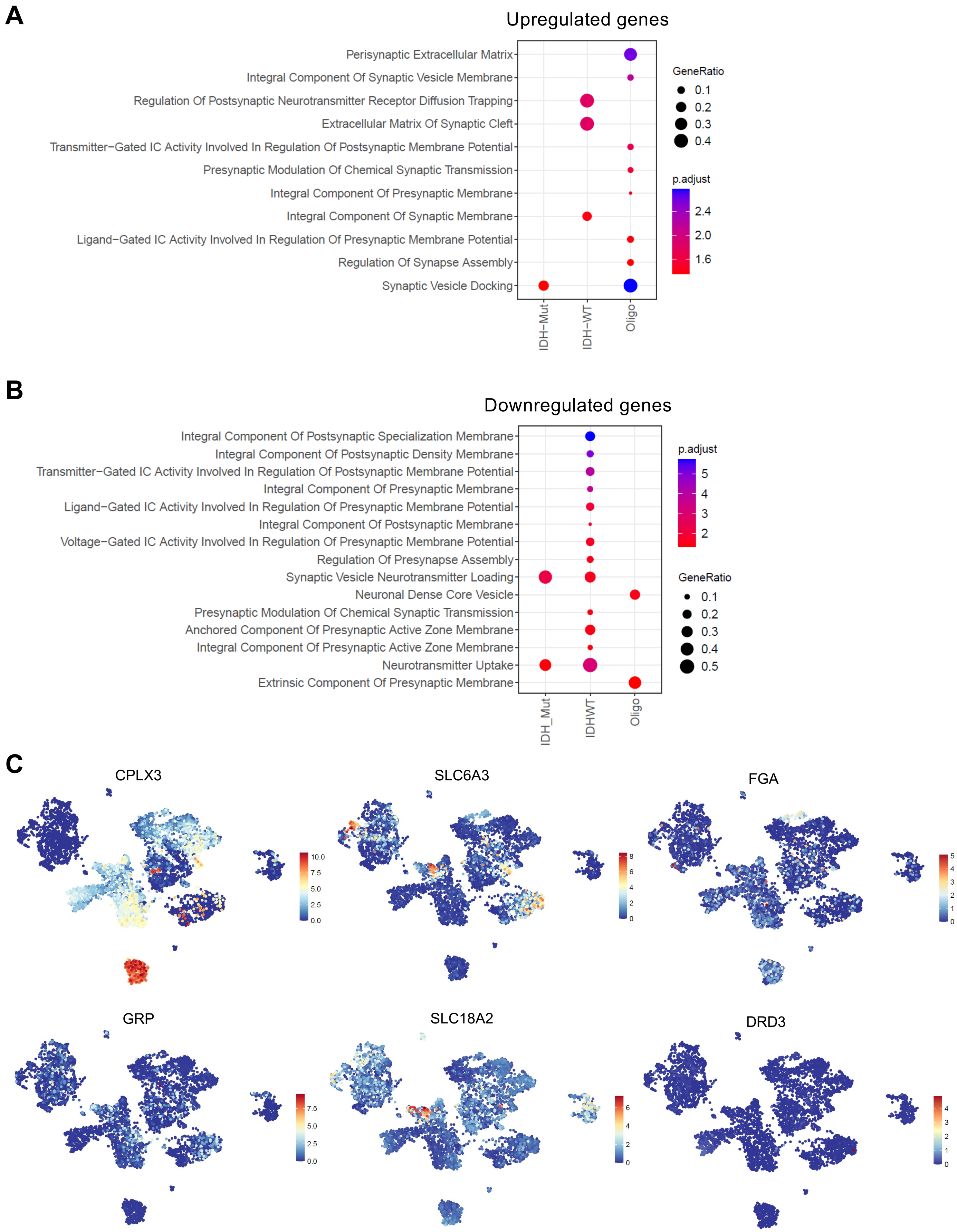

bioRxiv preprint doi: https://doi.org/10.64898/2026.09.30.755821; this version posted October 2, 2026. The copyright holder for this preprint (which was not certified by peer review) is the author/funder, who has granted bioRxiv a license to display the preprint in perpetuity. It is made available under a CC-BY 4.0 International license.

Supplementary Figure 4

A

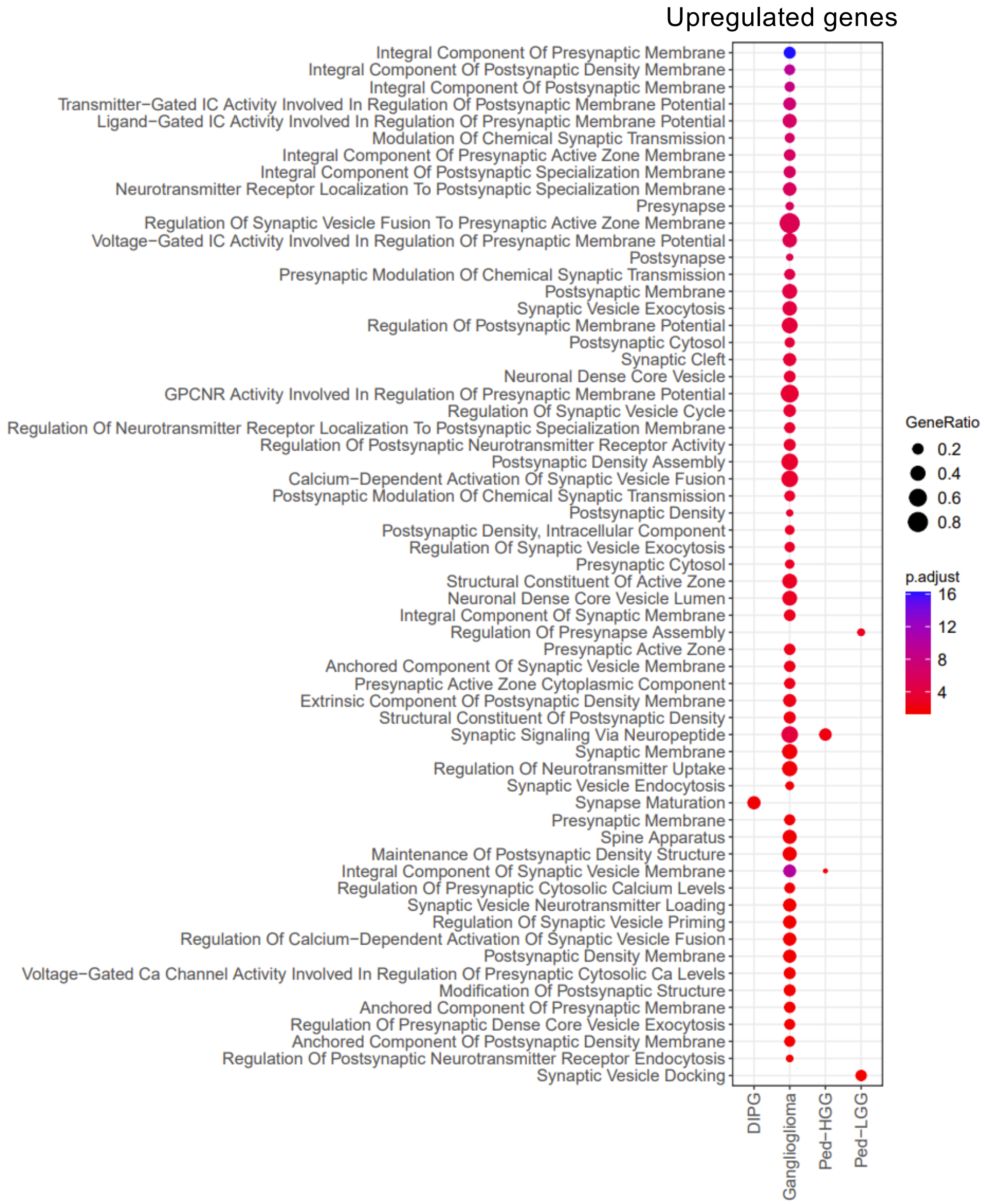

B

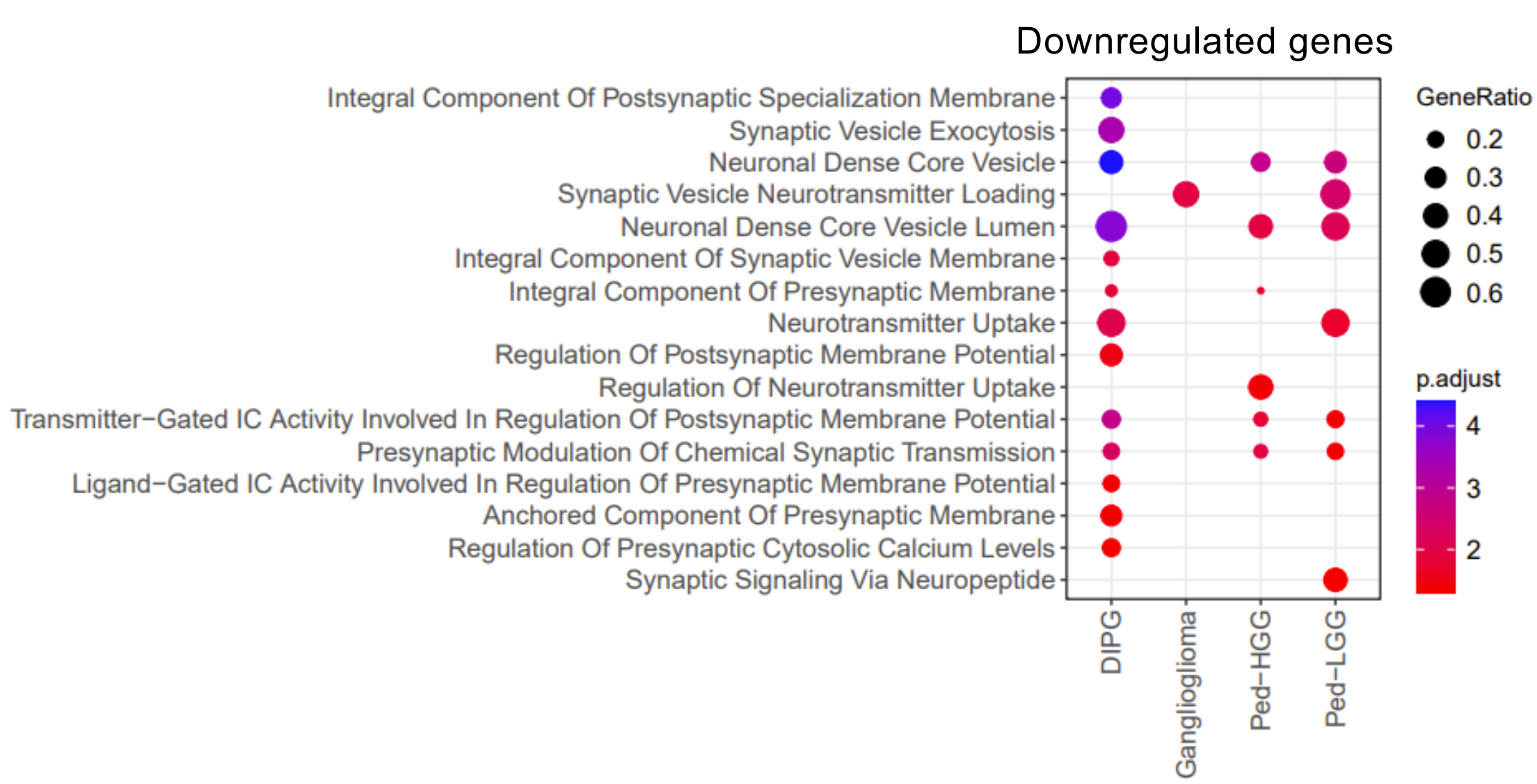

bioRxiv preprint doi: https://doi.org/10.64898/2026.09.30.755821; this version posted October 2, 2026. The copyright holder for this preprint (which was not certified by peer review) is the author/funder, who has granted bioRxiv a license to display the preprint in perpetuity. It is made available under a CC-BY 4.0 International license.

Supplementary Figure 5

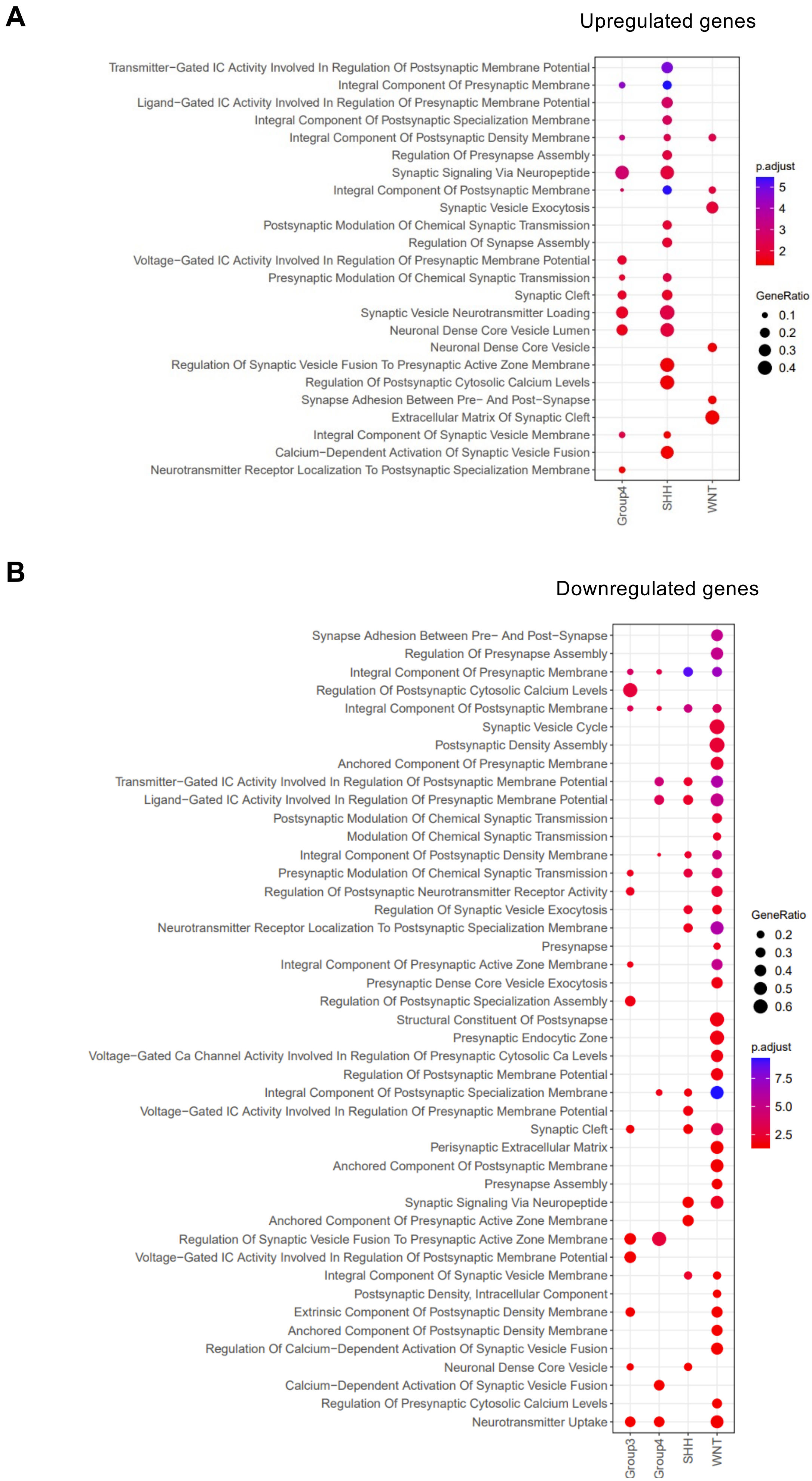

bioRxiv preprint doi: https://doi.org/10.64898/2026.09.30.755821; this version posted October 2, 2026. The copyright holder for this preprint (which was not certified by peer review) is the author/funder, who has granted bioRxiv a license to display the preprint in perpetuity. It is made available under a CC-BY 4.0 International license.

Supplementary Figure 6

A

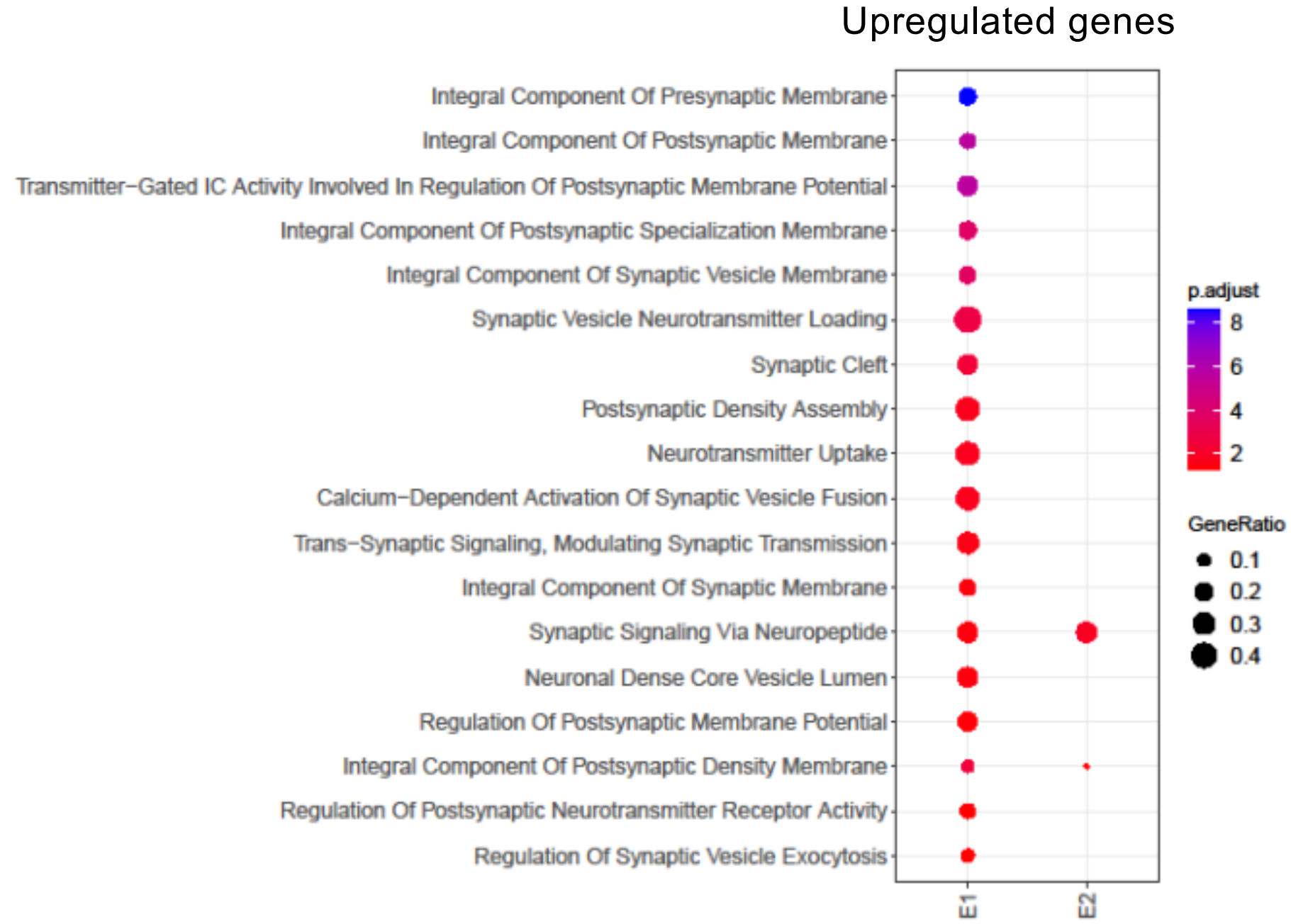

B

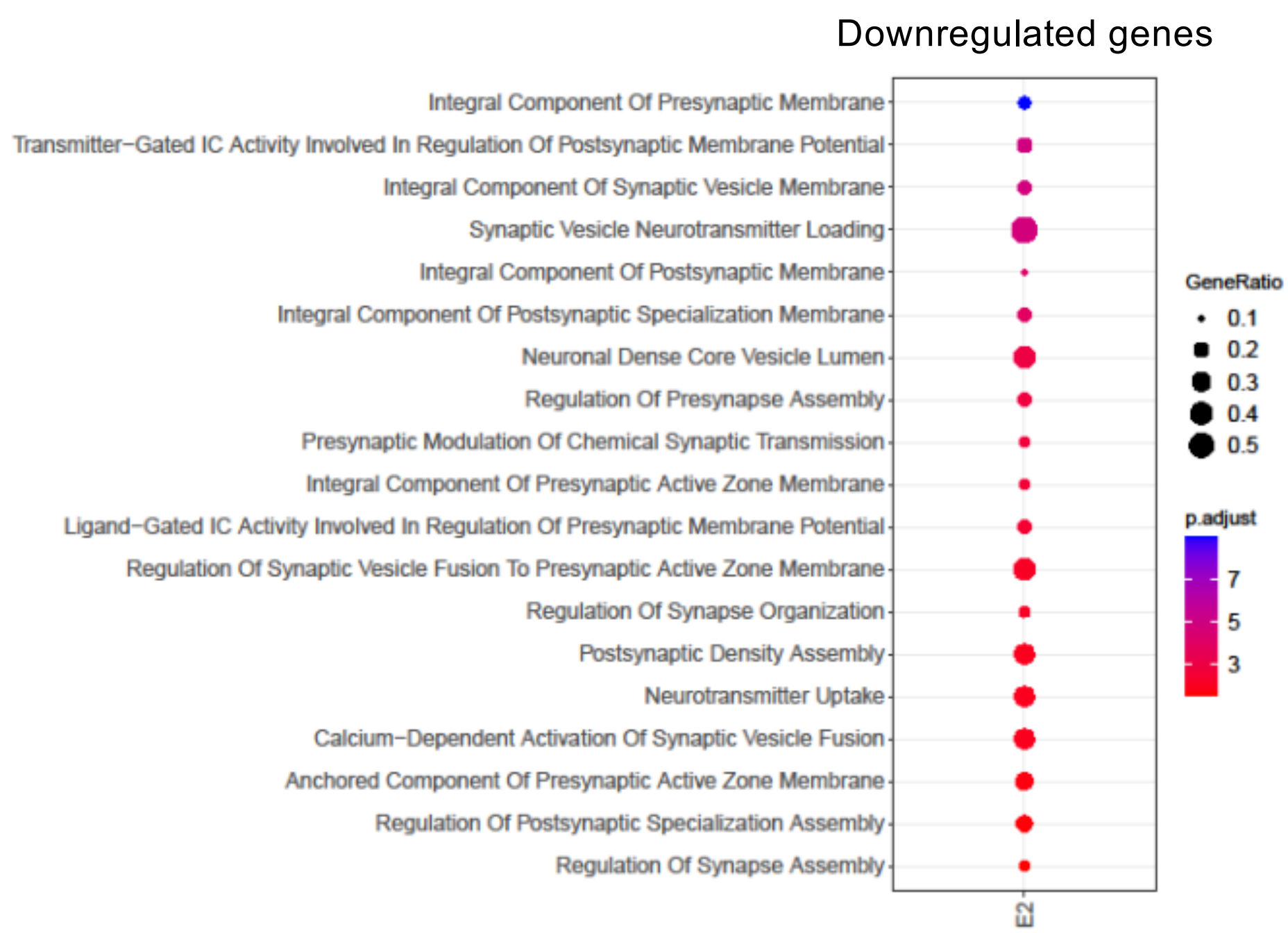

bioRxiv preprint doi: https://doi.org/10.64898/2026.09.30.755821; this version posted October 2, 2026. The copyright holder for this preprint (which was not certified by peer review) is the author/funder, who has granted bioRxiv a license to display the preprint in perpetuity. It is made available under a CC-BY 4.0 International license.

Supplementary Figure 7

A

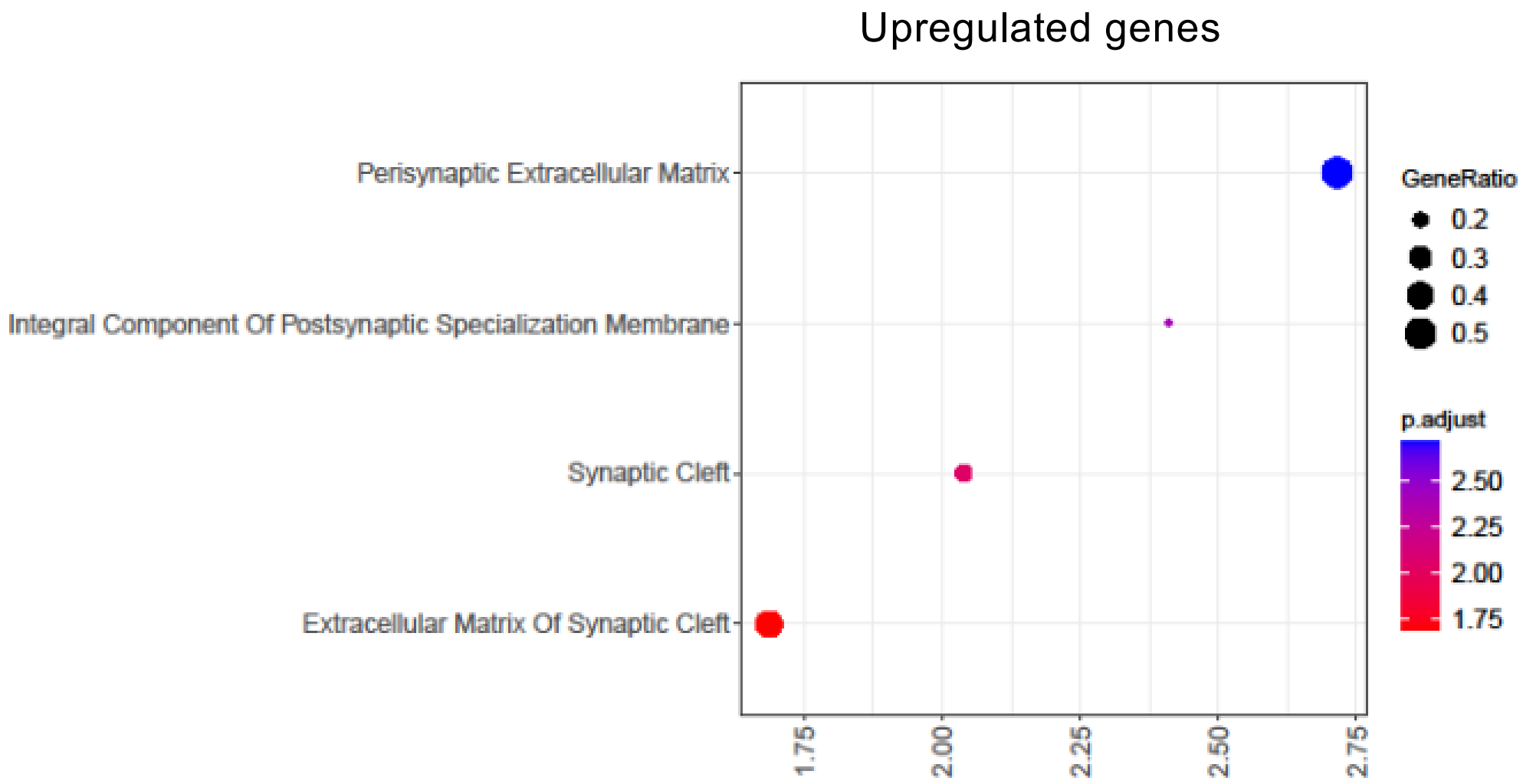

B

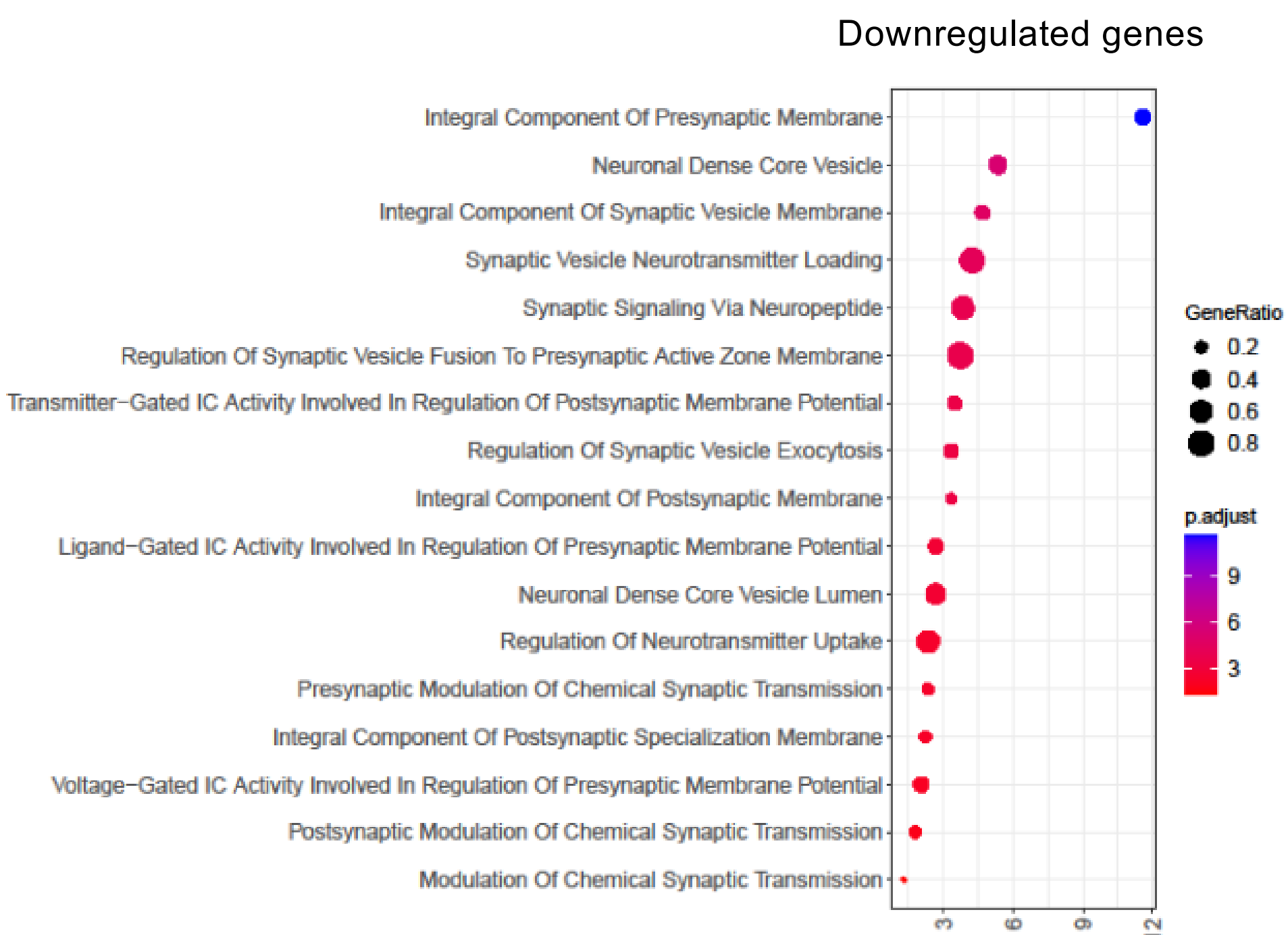

bioRxiv preprint doi: https://doi.org/10.64898/2026.09.30.755821; this version posted October 2, 2026. The copyright holder for this preprint (which was not certified by peer review) is the author/funder, who has granted bioRxiv a license to display the preprint in perpetuity. It is made available under a CC-BY 4.0 International license.

Supplementary Figure 8

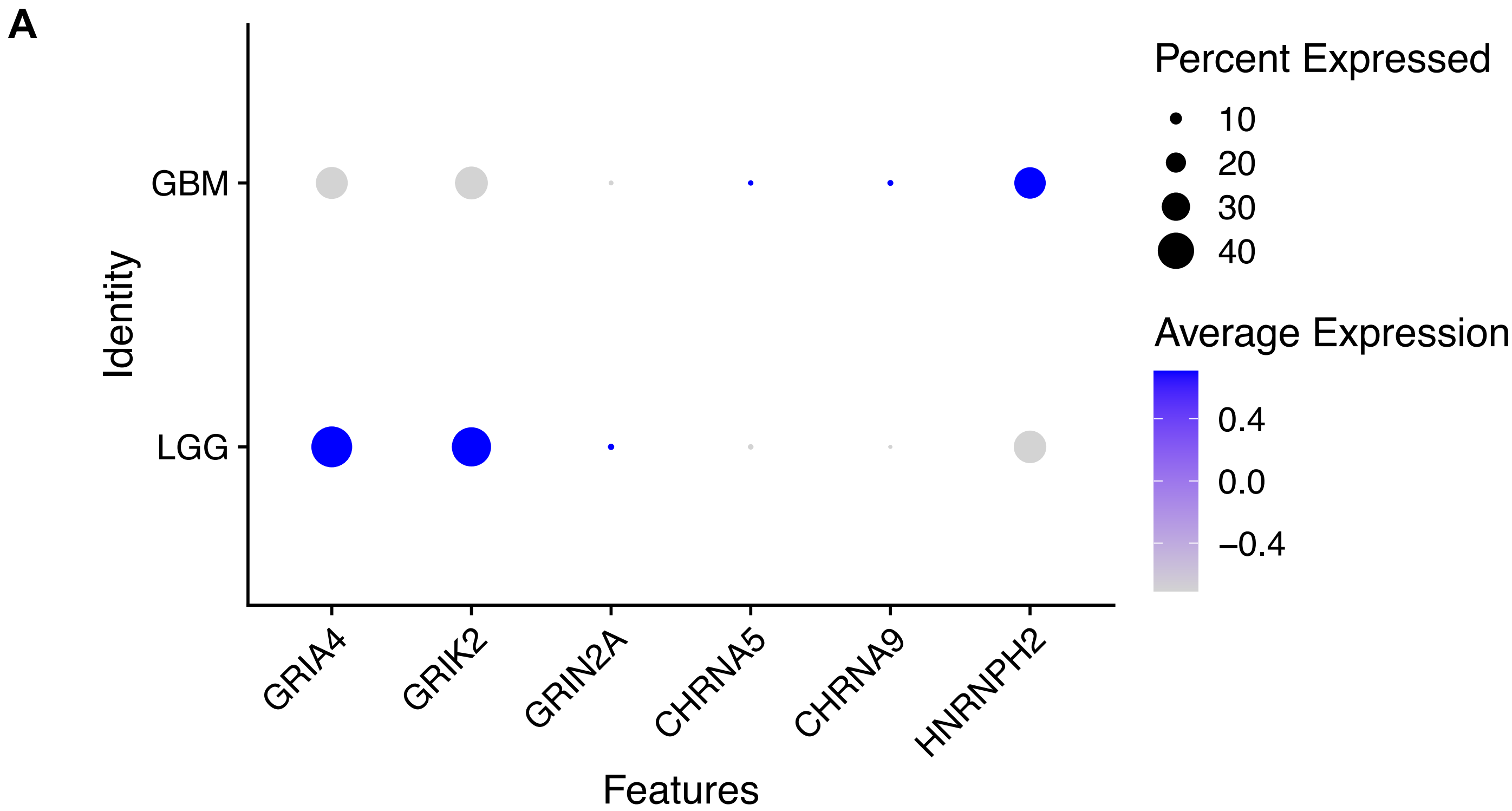

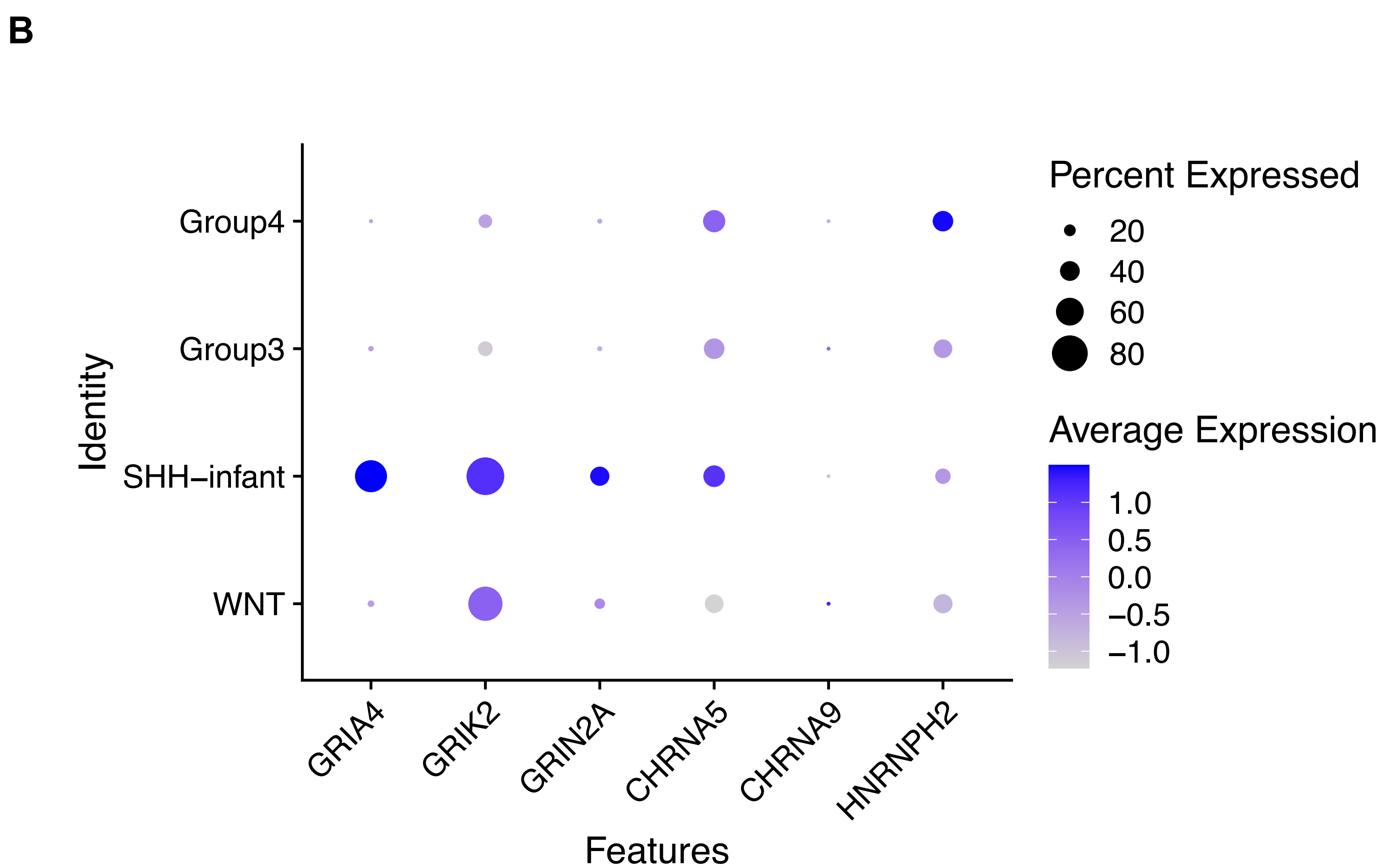

Supplement: Supplementary file 1 [file NIHPP2026.09.30.755821V1-supplement-1.pdf]
